# Electrically Reconfigurable Quasi‐BIC Metadevices Enabled by Integrated Barium Titanate

**DOI:** 10.1002/nap2.70104

**Published:** 2026-04-25

**Authors:** Jingyi Xia, Jietao Liu, Yi Liu, Zhi Liu, Xin Wei, Guofeng Song

**Affiliations:** ^1^ Institute of Semiconductors Chinese Academy of Sciences Beijing China; ^2^ College of Materials Science and Opto‐Electronic Technology University of Chinese Academy of Sciences Beijing China; ^3^ Institute of Intelligent Photonics Nankai University Tianjin China; ^4^ State Key Laboratory of Optoelectronic Materials and Devices Institute of Semiconductors Chinese Academy of Sciences Beijing China

**Keywords:** BaTiO_3_, BIC, electrical modulation, metadevices, optical metasurfaces, spectrometers

## Abstract

Quasi‐bound states in the continuum (quasi‐BIC, Q‐BIC) metasurfaces, endowed with their record high Q‐factor, promise paradigm‐shifts in applications of lasing, biosensing, and spectral filtering. However, their resonance is frozen, once the nanostructure is fixed, constraining real‐world deployability. We report an electrically reconfigurable quasi‐BIC metasurface that monolithically integrates barium titanate (BTO and BaTiO_3_) electro‐optic material and demonstrate its deployment as a high‐performance dispersion engine platform of a chip‐scale miniaturized intensity‐modulator and computational reconstructive spectrometer. By concurrently hosting a symmetry‐protected BIC and a guided‐mode resonance, the metasurface triggers an electromagnetically induced transparency (EIT) destructive interference in the near‐infrared, delivering a bandwidth of λ/2128 narrow band‐stop filter. Systematic analysis of structural parameters reveals mode coupling dynamics. Leveraging the Pockels effect and mode confinement in BTO, a linear tuning slope of 1.79 nm/V is achieved. Leveraging the metasurface as a voltage‐scanned tunable filter, we furtherly demonstrate a 250 μm^2^ miniaturized spectrometer. A spectral reconstruction optimization algorithm recovers spectra with 0.2 nm resolution, 99% fidelity, and 89.47% optical throughput. These results offer a technical pathway toward miniaturized spectral engines combining sub‐nanometer resolution with chip‐scale portability. A scalable platform is established for active metadevices with reconfigurable Q‐factor and spectral agility.

## Graphical abstract

An electrically reconfigurable quasi‐BIC metasurface is demonstrated by monolithic integration of barium titanate. Leveraging the Pockels effect, linear tuning of 1.79 nm/V is achieved, enabling a 250 μm^2^ miniaturized spectrometer with 0.2 nm resolution, 99% fidelity, and high throughput.

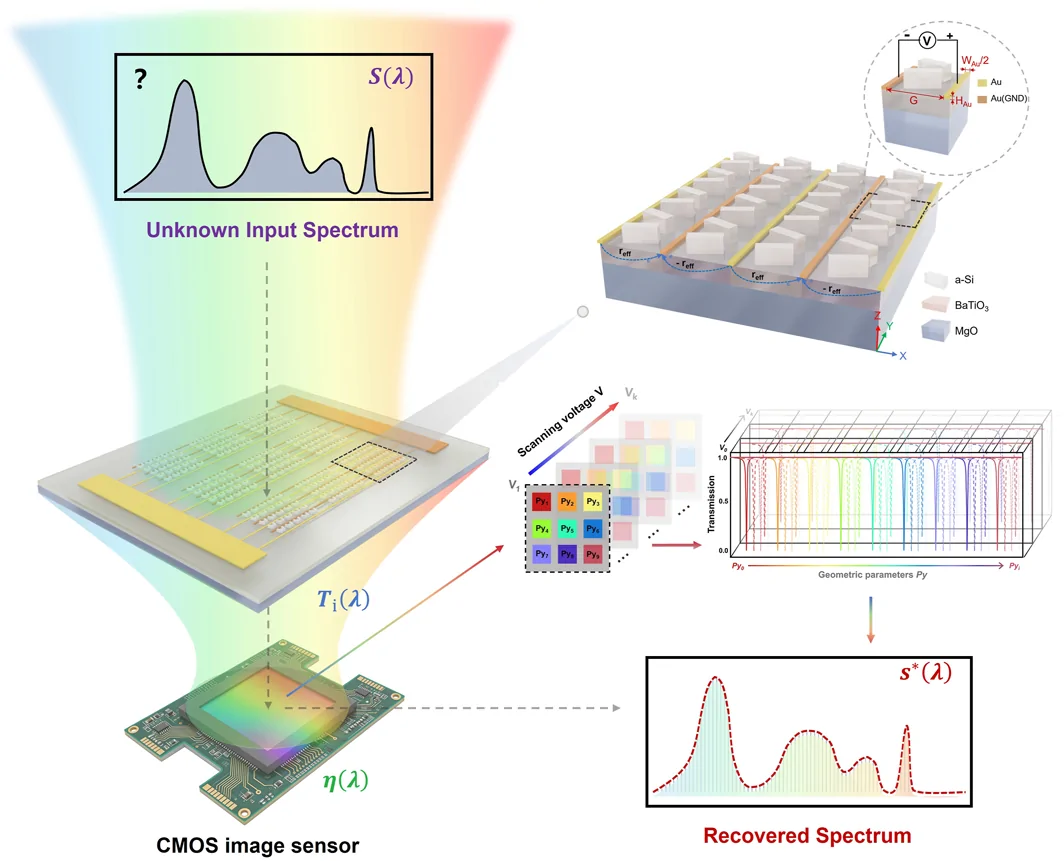

## 1 Introduction

Optical metasurfaces, two‐dimensional artificial micro/nanostructures, provide precise control over the amplitude, phase, and polarization of light, offering a scalable foundation for integrated photonics [1, 2, 3]. Among them, quasi‐BIC metasurfaces, which utilize bound states in the continuum, are at the forefront of research due to their ultra‐high quality‐factors and strong field confinement [4, 5, 6, 7, 8]. These attributes render them highly promising for biomolecular sensing [9, 10], enhanced nonlinear optics [11, 12], and narrowband filtering [13, 14]. However, the operation of most current quasi‐BIC metasurfaces demonstrated to date is governed by fixed geometric parameters, leading to a static resonant wavelength and profile that lacks dynamic reconfigurability at the moment of fabrication [15, 16]. Such passivity is adequate for sensing [17]; however, this static response is insufficient for advanced applications requiring active real‐time tuning, such as reconfigurable photonic devices and intelligent spectral analysis, thereby constraining their practical application scope.

To break the static limitations of metasurfaces, various active materials and dynamic tuning strategies have been explored [18]. Yet, every mainstream route carries a non‐negotiable drawbacks and penalty: microelectro‐mechanical systems (MEMS) enable mechanical tuning but suffer from slow response and structural complexity [19]; carrier‐based tuning using graphene or transparent conductive oxides provides high speed at the cost of high optical loss and significant Q‐factor degradation [20, 21]; phase‐change materials face challenges in switching speed, cycling stability, and integration [22]; liquid crystals exhibit slow millisecond‐scale responses and process compatibility issues [23]. In contrast, tuning via the Pockels effect in electro‐optic crystals, such as lithium niobate or BTO, combines nanosecond‐scale speed with low loss, showing excellent compatibility with high‐Q metasurfaces [24, 25, 26]. Notably, BTO has an electro‐optic coefficient 6–30 times larger than that of lithium niobate [27], offering an ideal platform for low‐power, highly sensitive, and easily integrable electrically tunable metadevices.

Among these tunable metadevices, compact miniaturized spectrometers as core components of on‐site detection and portable devices are highly demanded for higher performance [28, 29]. However, current metasurface‐based designs encounter significant scaling challenges. In computational spectroscopy, spectral diversity is usually harvested from large arrays of fixed‐response metasurfaces—submillimeter to centimeter‐scale footprints that erase the size advantage of flat/meta‐optics [30]. Meanwhile, systems based on static narrowband filter arrays face a fundamental trade‐off: improving spectral resolution generally sacrifices optical throughput, reducing the sensitivity and signal‐to‐noise ratio [31]. These constraints constitute a fundamental barrier to continued miniaturization and performance maximization. Consequently, the field demands spectral engines that simultaneously deliver high throughput, sub‐nanometer resolution and wafer‐scale compactness.

To address these challenges, we integrate the high‐performance electro‐optic crystal BTO with high‐Q quasi‐BIC metasurfaces to construct an electrically tunable narrowband filter. This approach simultaneously tackles dynamic tuning, sub‐nanometer high resolution, and true chip‐scale footprint. By leveraging the strong field localization of the quasi‐BIC mode, the design achieves narrowband band‐stop filtering, enabling optical throughput to scale with spectral resolution—thereby breaking the inherent resolution–sensitivity trade‐off of traditional systems [32]. Furthermore, using the inherent high Pockels coefficient of BTO, rapid and precise resonance tuning is achieved via bias voltage control, significantly reducing the scale of conventional static filter arrays and improving integration [33]. Full‐wave simulations show that the BTO metasurface unit attains a 0.7 nm bandwidth and an electrical tuning sensitivity of 1.8 nm/V. The resulting miniaturized spectrometer, with a core size of only ≈ 250 μm^2^, achieves 0.2 nm spectral resolution and over 99% average reconstruction fidelity while maintaining high throughput. This work presents a new design paradigm for tunable metasurfaces and showcases the BTO–quasi‐BIC platform's potential for integrated photonic devices [34, 35].

## 2 Metasurface Design

To achieve high‐Q resonance, the metasurface is designed as a three‐layer waveguide (Air/BTO/MgO) with a periodic amorphous silicon (*a‐Si*) rod‐pair layer (see Figure 1a). This simple high‐index‐contrast waveguide supports guided modes (GM). By introducing the periodic *a‐Si* nanostructures perturbation layer, the GM odd mode evolves into a symmetry‐protected bound state in the continuum (BIC), yielding a theoretical infinite Q‐factor; whereas the even mode becomes a leaky guided‐mode resonance (GMR) with a finite Q [36]. The results in the Figure 1b,c depict the top and side views of one unit cell. The key geometric parameters are designed as follows: periods *P*_*x*_ = 700 nm and *P*_*y*_ = 750 nm; *a‐Si* rod length *a* = 350 nm, width *b* = 120 nm, center‐to‐center distance *d* = 340 nm (along the *Y‐axis*), orientation angle *θ* = 15° relative to the *X*‐axis, and the height *H*_*a‐Si*_ = 180 nm; and the BTO film thickness *H*_BTO_ = 300 nm. To circumvent the substantial sidewall roughness associated with etching BTO using current micro‐nanofabrication techniques [37], our design avoids patterning the BTO layer. Simulations are performed using the finite element method (FEM, with COMSOL Multiphysics) and finite‐difference time‐domain (FDTD) method. Detailed material parameters and the simulation settings are provided in the Supporting Information S1: Note S1.

**FIGURE 1:**
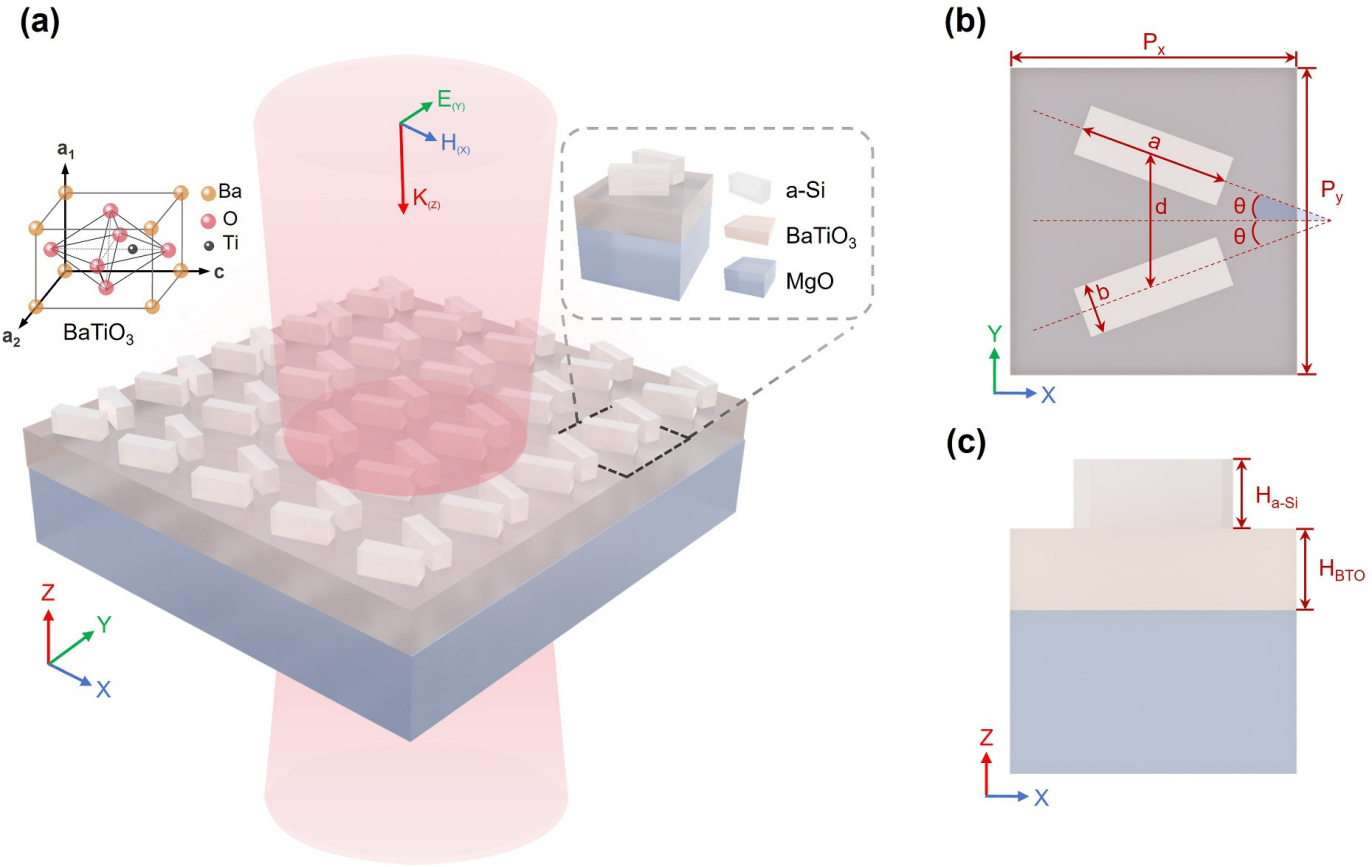
Metasurface architecture. (a) Schematic of the BTO metasurface. (b) Top view of one unit cell (*X–Y* plane) of the periodic array. (c) Side view of the unit cell (*X–Z* plane).

When illuminated vertically by transverse electric (TE)‐polarized light in the near‐infrared band, multiple narrow‐linewidth resonance peaks appear in the transmission spectrum. The results are shown in Figure 2a,b. The eigenmodes of the structure are analyzed by calculating the photonic band structure and Q‐factors. The finite Q‐factors at the *Γ* point confirm that the TE modes at wavelengths of 1371.7 nm (GMR_1_) and 1382.9 nm (GMR_2_) are the GMR modes, meanwhile a Q‐factors exceeding 10^6^ at the *Γ* point confirm the quasi‐BIC modes at 1383.9 nm (Q‐BIC_2_) and 1488.2 nm (Q‐BIC_1_) transformed from the BIC modes as detailed in the Supporting Information S1: Note S2. As shown in the Figure 2b, we further performed numerical simulations of the transmission spectrum near the Q‐BIC_1_ mode using FDTD method. The FDTD result is consistent with the FEM result, confirming the accuracy of simulations.

**FIGURE 2:**
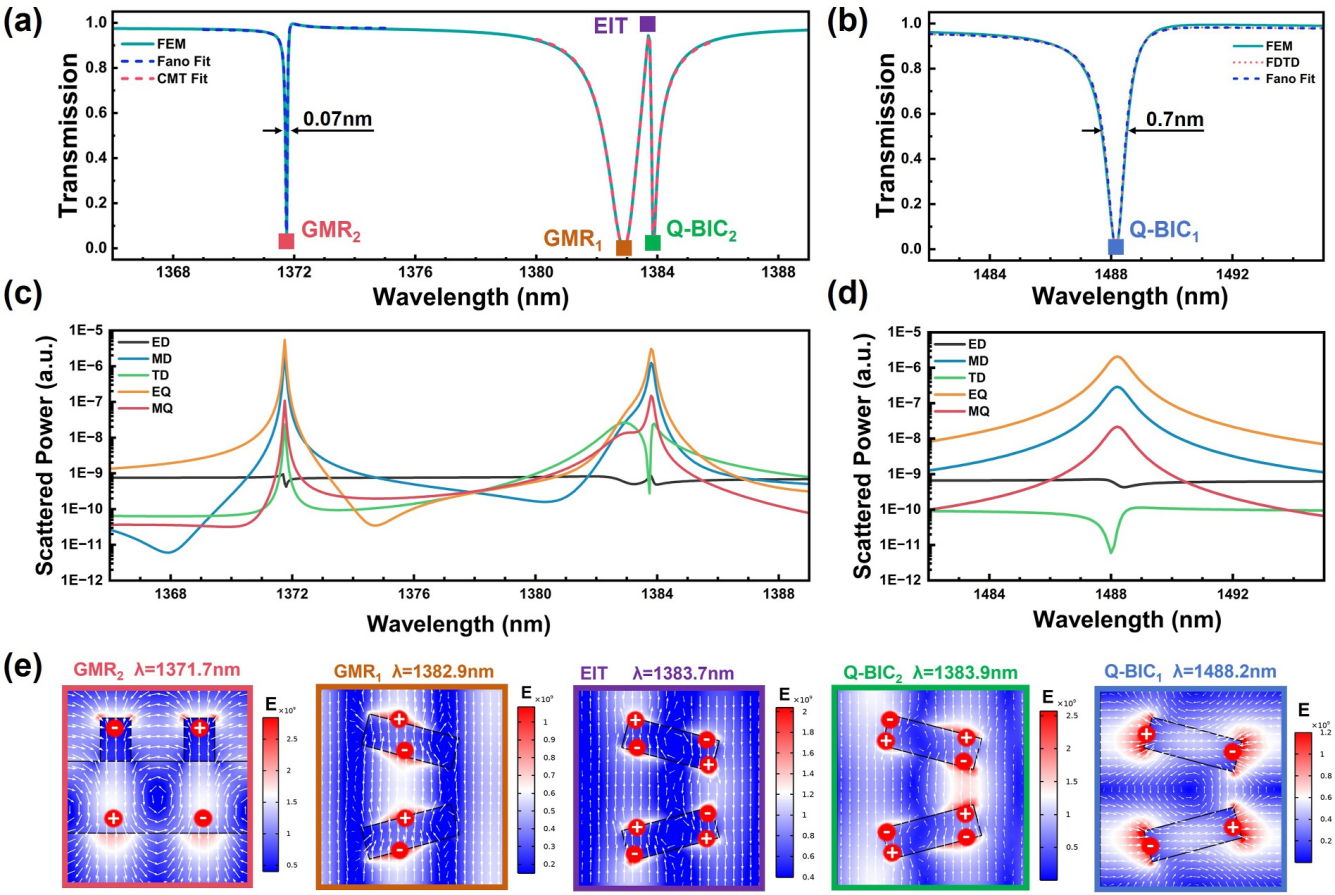
Optical response of the BTO metasurface. (a, b) Transmission spectra: cyan solid line (FEM), blue‐dashed curve (Fano fit), orange‐dashed line (CMT fit), and orange dotted line (FDTD). (c, d) Corresponding multipole decomposition of the scattering cross‐section. (e) Electric field distributions (*|E|*) at the five resonances positions. The white arrows indicate the in‐plane electric field vectors. The ± symbols mark the centers of positive and negative bound charges forming the electric dipole (ED) moments.

Additionally, the Q‐factors of these GMR and Q‐BIC modes are analyzed. The GMR_2_ and Q‐BIC_1_ modes (see Figure 2a,b) are estimated by fitting the transmission spectrum with a Fano profile formula [38],

(1) $$T\left(\omega \right)=\left|T_{0}+A_{0}\frac{{\left[q+2\left(\omega ‐\omega_{0}\right)/\Gamma \right]}^{2}}{{1+\left[2\left(\omega ‐\omega_{0}\right)/\Gamma \right]}^{2}}\right|,$$

here $T_{0}$ represents the background scattering parameter, $A_{0}$ denotes the coupling coefficient, q is the Fano parameter that determines the asymmetry of the resonant profile, $\omega_{0}$ is the resonant frequency, and Γ is the full width at half maximum (FWHM) of the resonance. The Q‐factor is calculated via $Q=\omega_{0}/\Gamma$. As indicated by the blue dashed curves in the Figure 2a,b, the Fano fitting agrees perfect with the FEM results. The calculated FWHM values for the GMR_2_ and the Q‐BIC_1_ modes are 0.07 and 0.7 nm, corresponding to Q‐factors of 19600 and 2128, respectively. Furthermore, we have supplemented the transmission spectral response and analysis of the proposed metasurface under TM mode as detailed in Supporting Information S1: Note 3.

It is noteworthy that an EIT window is observed between GMR_1_ and Q‐BIC_2_ (see purple marker in Figure 2a). EIT with sharp transmission spectral features holds significant applications in optical switching, sensing, and slow‐light devices [39]. To gain deeper insight into this EIT effect, we employ the coupled‐mode theory (CMT) to analyze the coupling between these two modes (GMR_1_ and Q‐BIC_2_). The transmission amplitude *t(ω)* of the coupled system is described as follows (detailed CMT calculation is provided in the Supporting Information S1: Note S4),

(2) $$t\left(\omega \right)=1-\frac{\gamma_{1r}}{j\left(\omega ‐\omega_{1}\right)+\gamma_{1}+\frac{\kappa^{2}}{j\left(\omega ‐\omega_{2}\right)+\gamma_{2}}},$$

here $\omega_{1}$ and $\omega_{2}$ represent the resonant frequencies of GMR_1_ and Q‐BIC_2_ modes, respectively; $\gamma_{1}$ and $\gamma_{2}$ denote their total loss rates, where $\gamma_{i}$ = $\gamma_{ir}+\gamma_{ia}$. $\gamma_{ir}$ represents the radiative loss, and $\gamma_{ia}$ the absorption loss, whereas κ is the coupling coefficient between GMR_1_ and Q‐BIC_2_ modes. The transmittance is given by $T\left(\omega \right)={\left|t\left(\omega \right)\right|}^{2}$. As shown by the pink‐dashed curve in Figure 2a, the CMT fitting agrees almost perfect with the FEM result. The extracted parameters are $\gamma_{1}$ = 7.09 × 10^11^ rad/s, $\gamma_{2}$ = 2.91 × 10^9^ rad/s, and κ = 3.45 × 10^11^ rad/s. The calculated coupling efficiency is $\eta =\kappa /\sqrt{\gamma_{1}\gamma_{2}}$ = 7.6 >> 1, indicating strong coupling between the two distinct modes when their resonant frequencies approach each other. In this regime, energy transfers from the high‐loss GMR_1_ bright mode to the low‐loss Q‐BIC_2_ dark mode. Due to the extremely low radiative loss of Q‐BIC_2_, the energy is temporarily trapped. When this energy recouples back into the bright mode, destructive interference occurs at a specific frequency, resulting in the extremely narrow transparency window within the original transmission dip of the GMR.

To gain deeper understanding of the origin of the resonances in the spectrum, multipole expansion analysis is performed [40] and the corresponding electric field distributions at the resonance positions are shown in the Figure 2c–e. The GMR_2_ mode exhibits strong field localization within the BTO layer, characterized by an electric dipole (ED) in each *a‐Si* rod and the BTO beneath it. Across the unit cell, a pair of these antiparallel EDs forms an overall electric quadrupole (EQ) moment, which strongly suppresses the radiation coupling, leading to an extremely narrow resonance linewidth of the mode. The electric field distributions show that GMR_1_, the EIT window, and Q‐BIC_2_ are all associated with an electric quadrupole (EQ) in the *a‐Si* rods. The GMR_1_ and Q‐BIC_2_ exhibit localized fields; the EIT window shows a delocalized profile. The Q‐BIC_1_ mode, in contrast, arises from a pair of antiparallel EDs on the two rods, forming an overall EQ across the unit cell. The EIT phenomenon is typically accompanied by a slow light effect due to the strong dispersion within the transparency window. We calculated the group time delay (T_g_), which characterizes the slow light effect, and the results indicate a group time delay of 11.2 ps at the EIT transparency window. Details are provided in Supporting Information S1: Note 5.

The influence of various geometric parameters on the transmission spectrum of the designed metasurface is analyzed in Figure 3a–e. By increasing the geometrical dimensions, *a* and *b* of the *a‐Si* rods and the period *P*_*y*_, all resonant wavelengths redshift to long‐wavelength as a result of the increased unit cell volume and elevated effective refractive index. Remarkably, a decrease in *a* and *b* specifically leads to a distinct anticrossing (Rabi splitting) of these two GMR_1_ and Q‐BIC_2_ resonance modes (see colored belts depicted in the Figure 3a,b), a feature of strong coupling. Benefiting from the strong field localization in the BTO layer, the Q‐BIC_1_ mode exhibits a resonant wavelength highly sensitive to the lattice period *P*_*y*_ but insensitive to the orientation angle *θ*. This enables a decoupled tuning: by varying *θ*, we can adjust the Q‐factor while keeping the resonant wavelength slightly‐changed; whereas stretching *Py* shifts the wavelength while leaving the Q‐factor intact (see Figure 3c). This independent control of position and linewidth of resonance is ideal for designing metasurface arrays as miniaturized pixelated spectrometers. The resonance peaks of Q‐BIC_1_ and Q‐BIC_2_, see Figure 3d,e, are sharpened with decreasing orientation angle *θ* and vanishes at *θ* = 0°. Additionally, Figure 3f shows that the Q‐factor of Q‐BIC_1_ scales as 1/*α* ^2^ (with the asymmetry parameter *α* =sin(θ)). This behavior is typical characteristic of the symmetry‐protected BIC [41].

**FIGURE 3:**
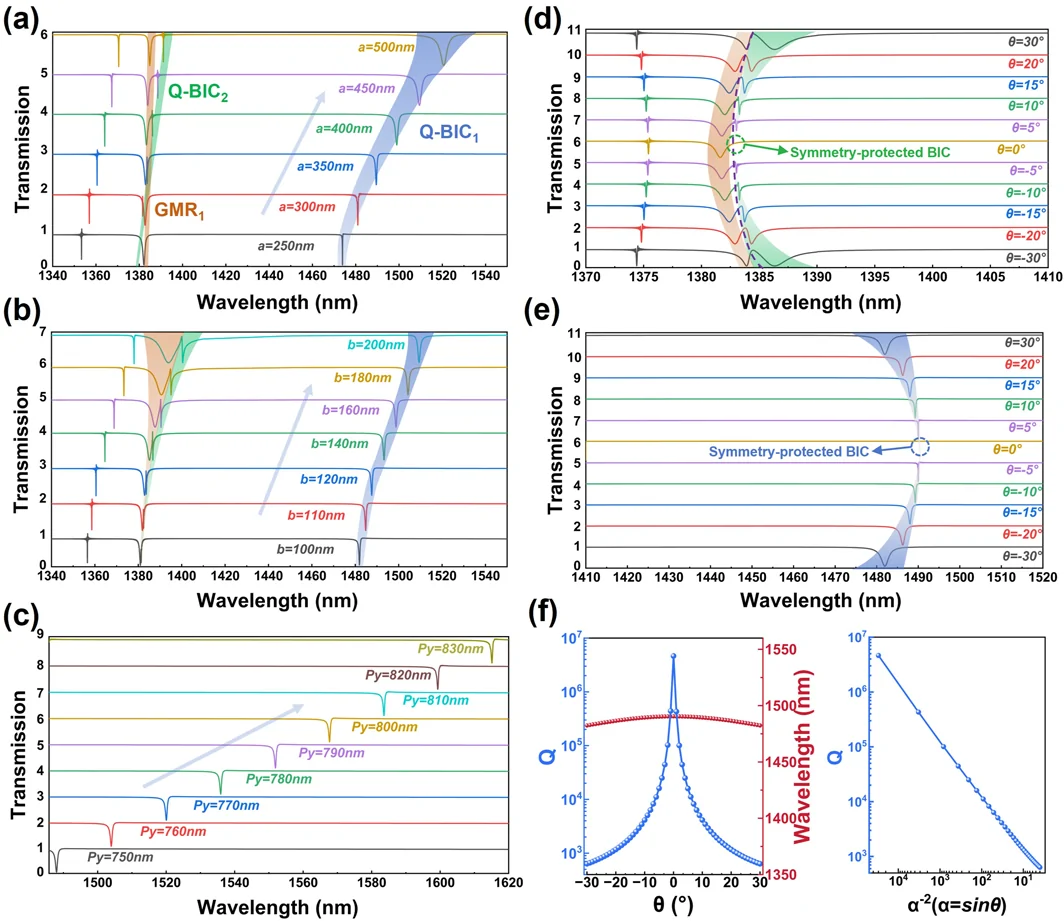
Transmission spectra of the metasurface as a function of varied geometric parameters. (a–e) Transmission spectra under different values of *a*, *b*, *P*_*y*_, and *θ*. (f) The dependence of the Q‐BIC_1_ mode's Q‐factor and resonant wavelength on orientation angle *θ* and the asymmetry parameter *α* (α=sin(θ)).

Despite fine tunability can be obtained from geometric and topological design of the metasurface, active and efficient control with reconfigurability is highly desired. Therefore, too achieve dynamic active tuning, we exploit the Pockels effect in BTO to shift the resonant wavelength with an voltage applied. This linear electro‐optic effect, characteristic of noncentrosymmetric crystals BTO, induces a refractive index change proportional to the electric field. The relationship can be expressed as follows [42]:

(3) $$\Delta n\left(\varphi \right)=\frac{1}{2}n^{3}r_{\text{eff}}\left(\varphi \right)\Gamma \left(\varphi \right)E,$$

where Δn is the induced refractive index change in BTO, $r_{\text{eff}}\left(\varphi \right)$ is the effective electro‐optic coefficient, and E=V/G (with applied voltage V and electrode gap G). The term Γ(φ) represents the electric‐optical overlap factor. Here, φ denotes the angle between the optical axis of BTO crystal and the applied electric field E. The parameter n is the intrinsic refractive index of BTO (zero field). The expressions for $r_{\text{eff}}\left(\varphi \right)$ and Γ(φ) used in this work are given as follows [43, 44]:

(4) $${\;r}_{\text{eff}}\left(\varphi \right)=r_{33}\cos^{3}\left(\varphi \right)+\left(r_{13}+2r_{51}\right)\sin^{2}\left(\varphi \right)\cos\left(\varphi \right),$$

(5) $$\Gamma \left(\varphi \right)=\frac{1}{E_{A}}\;\frac{\int_{\text{BTO}}E_{e}\left(\varphi \right){\left|E_{op}\left(\varphi \right)\right|}^{2}dV_{\text{BTO}}}{\int_{\text{BTO}}{\left|E_{op}\left(\varphi \right)\right|}^{2}dV_{\text{BTO}}},$$

here based on the experimental study and data by *Stefan Abel* et al. [45], we use the three primary electro‐optic coefficients of BTO as $r_{13}=10\text{pm}/V$, $r_{33}=342\text{pm}/V$, and $r_{51}=r_{42}=923\text{pm}/V$. According to the Equation (4), to maximize $r_{\text{eff}}\left(\varphi \right)$, we set *φ* = 50°, the calculated effective electro‐optic coefficient is $r_{\text{eff}}=r_{\text{eff}}\left(50{}^{\circ}\right)=791\text{pm}/V$. To evaluate Γ(φ) more rigorously, we introduce the electric field per unit volume, $E_{A}$,

(6) $${\;E}_{A}=\frac{\int_{\text{BTO}}\left|E_{V}\right|dV_{\text{BTO}}}{V_{\text{BTO}}},$$

where $E_{V}$ is the electric field generated by the applied voltage and $V_{\text{BTO}}$ represents the volume of the BTO region. Details of the analysis of Γ(φ) and *φ* are provided in Supporting Information S1: Note S6.

For dynamic Pockels‐effect tuning, interdigitated Au electrodes are incorporated into the unit cell design (see Figure 4a), with an electrode width *W*_*Au*_ = 60 nm, height *H*_*Au*_ = 30 nm, and an electrode gap *G*. Our supplementary analysis shows that the introduction of the gold electrodes has a minimal impact on the Q‐BIC_1_ resonance peak. Therefore, the subsequent demonstration of the electrically reconfigurable quasi‐BIC metasurface is primarily based on the Q‐BIC_1_ mode (see Supporting Information S1: Note S7 for details). The BTO crystal orientation is defined by post‐fabrication poling. Under an applied voltage, this electrode configuration will create a periodic sign alternation in the effective electro‐optic coefficient $r_{\text{eff}}$ across successive periods. The active electrical tunability of the BTO metasurface is demonstrated by calculating the transmission spectra under voltages from −10 to +10 V for the Q‐BIC_1_ resonance at *P*_*y*_ = 780 nm as shown in the Figure 4b. The resonance peak red‐shifts with increasing voltages, yielding a central‐wavelength tuning sensitivity of ∼1.79 nm/V. As shown in Figure 4c, the wavelength tuning follows a pleasant linear trend (λ=1.79V+1539), whereas the modulation depth, defined as $M=\frac{T_{\text{background}}-T_{\min}}{T_{\text{background}}}\times 100\%$, remains high above 90% and reaches a maximum of 93.5%. Currently, barium titanate (BTO)‐based electro‐optic modulators can operate at frequencies ranging from 5 MHz to 110 GHz [46, 47, 48], corresponding to a response time on the nanosecond scale. Leveraging this high‐speed capability along with our metasurface design enables the realization of multichannel intensity modulators that operate at high speed and with low power consumption. As shown in Figure 4d, at a wavelength of 1550 nm, the device requires only a 0.8 V swing to deliver successive intensity modulation with a depth of 93.17%, while maintaining a broad linear response exceeding 70%.

**FIGURE 4:**
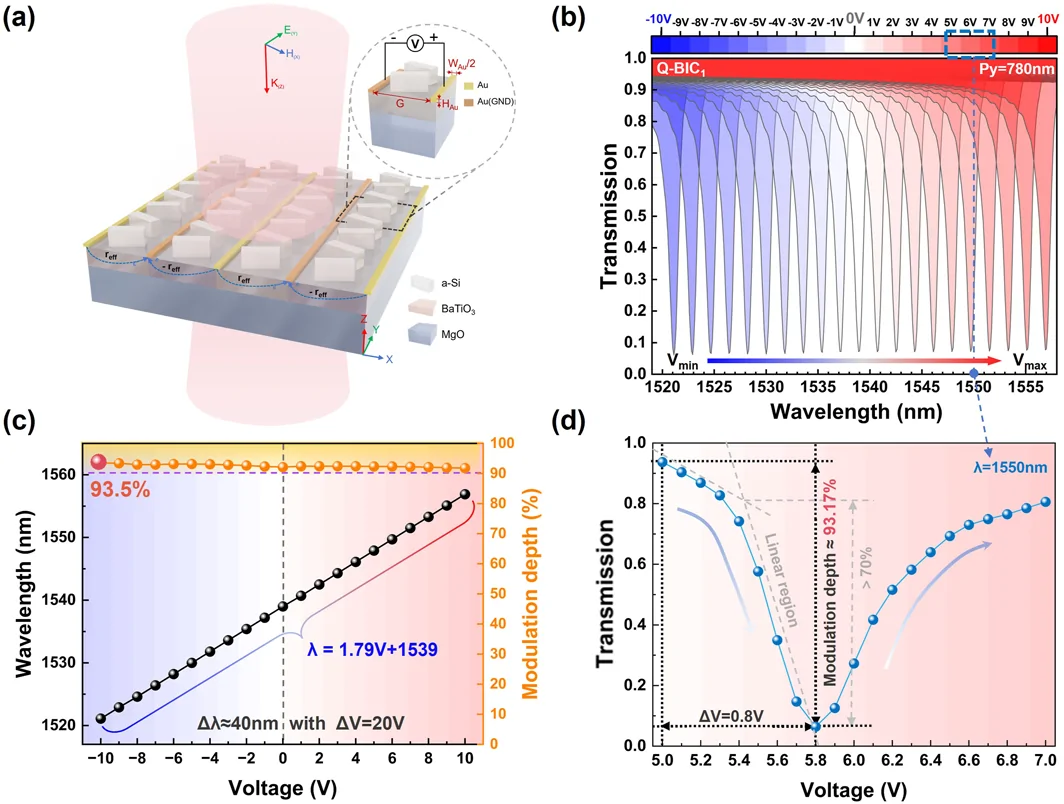
Active dynamic tunability of the metasurface. (a) Schematic of metadevices with interdigitated electrodes. The blue dotted‐arrows in BTO‐film denote the applied field direction. (b) Evolution of the Q‐BIC_1_ transmission spectra with voltages applied (−10 to +10 V, with 1 V step) at a fixed period *P*_*y*_ = 780 nm. (c) Dependence of wavelength and the modulation depth as a function of voltages applied. (d) Variation of transmission with voltage (intensity‐modulator) for Q‐BIC centered at 1550 nm.

We analyze the metasurface spectrum to map each resonance to its mode and elucidate the strong‐coupling nature of the EIT window. Systematic studies of geometric (static and fine) and electrical (active and dynamic) tunability are presented. These results establish a unified tuning framework and define a class of agile metadevices for reconfigurable photonics.

## 3 Miniaturized Spectrometer Metadevices Based on the Barium Titanate Metasurfaces

Bulky optics and precision mechanics render conventional spectrometers costly, fragile, and field‐incompatible. Although metasurface‐based miniaturized spectrometers offer a promising chip‐scale alternative, those relying on static narrowband filter arrays face a fundamental trade‐off: higher spectral resolution reduces optical throughput and sensitivity. Seriously, covering a broad bandwidth requires numerous filter units, hindering further miniaturization. Our electrically tunable BTO metasurface overcomes these challenges by providing narrow band‐stop resonances with both static fine tunability and dynamic active control. This high‐throughput tunable platform enables highly sensitive and compact spectral detection.

To showcase miniaturized spectroscopy, we implement a compact 3 × 3 metasurface array (see Figure 5) and reconstruct spectra across 1450–1750 nm (300 nm spectral range). The array consists of 9 pixels with varied periods *P*_*y*_, each measuring 50 μm^2^ and spaced 25 μm apart, form a compact 250 μm^2^ spectrometer footprint. By scanning the applied bias voltage, a transmission matrix $T_{i}\left(\lambda \right)=\left[\begin{array}{cccc} T_{i,1}\left(\lambda \right) & T_{i,2}\left(\lambda \right) & \text{⋯} & T_{i,k}\left(\lambda \right) \end{array}\right]$ is constructed, where *i* denotes the *i*th metasurface pixel and *k* represents the *k*th voltage state. This transmission matrix $T_{i}\left(\lambda \right)$ can be precalibrated experimentally. The metasurface array is then integrated onto a CMOS image sensor. The incident light with an unknown spectrum S(λ) is encoded by the metasurface array and then detected by the CMOS sensor, generating a photocurrent response $I=\left[\begin{array}{ccc} I_{1} & \text{⋯} & I_{i} \end{array}\right]$. The photocurrent response $I_{i}$ of each metasurface pixel can be described by Simpson's integration formula [49]^,^

(7) $$I_{i}=\int_{\lambda_{\min}}^{\lambda_{\max}}S\left(\lambda \right)T_{i}\left(\lambda \right)\eta \left(\lambda \right)d\lambda ,$$

where η(λ) represents the relative quantum efficiency of the CMOS sensor. To reconstruct the incident spectrum S(λ), the problem is discretized and transformed into solving for the spectral vector $S\left(\lambda \right)={\left[\begin{array}{ccc} s\left(\lambda_{1}\right) & \text{⋯} & s\left(\lambda_{j}\right) \end{array}\right]}^{T}$,

(8) $$\left[\begin{array}{c} I_{1}\\ I_{2}\\ \text{⋮}\\ I_{i} \end{array}\right]=\left[\begin{array}{ccc} a_{11} & \text{⋯} & a_{1j}\\ \text{⋮} & \text{⋱} & \text{⋮}\\ a_{i1} & \text{⋯} & a_{ij} \end{array}\right]\times \left[\begin{array}{c} s\left(\lambda_{1}\right)\\ s\left(\lambda_{2}\right)\\ \text{⋮}\\ s\left(\lambda_{j}\right) \end{array}\right]$$

(9) $$a_{ij}=\int_{\lambda_{s}}^{\lambda_{\max}}T_{i}\left(\lambda \right)\phi_{j}\left(\lambda \right)d\lambda ,$$

here j denotes the number of spectral sampling points and $a_{ij}$ represents the elements of the coefficient matrix *A*. In this study, we employ piecewise polynomials [50] as the basis functions $\phi_{j}\left(\lambda \right)$. To address the ill‐posed nature of the spectral reconstruction problem, a regularized least squares method is employed. The reconstructed spectrum can be obtained by solving an optimization problem,

(10) $$s^{*}\left(\lambda \right)=\arg\min_{s\geq 0}{\left\|As\left(\lambda \right)-I\right\|}_{2}^{2}+\alpha {\left\|S\left(\lambda \right)\right\|}_{1}+\beta {\left\|S\left(\lambda \right)\right\|}_{2}^{2}+\gamma {\left\|DS\left(\lambda \right)\right\|}_{2}^{2}\;\left(10\right),$$

where $s^{*}\left(\lambda \right)$ is the reconstructed spectrum obtained through the computational reconstruction algorithm; α, β, and γ are the regularization parameters for sparsity, stability, and smoothness, respectively; D is the first‐order difference operator as a smoothing matrix used to enforce the spectral continuity constraint; and s≥0 ensures the physical non‐negativity of the spectral intensity. The flow of the reconstruction process is shown in the Figure 5.

**FIGURE 5:**
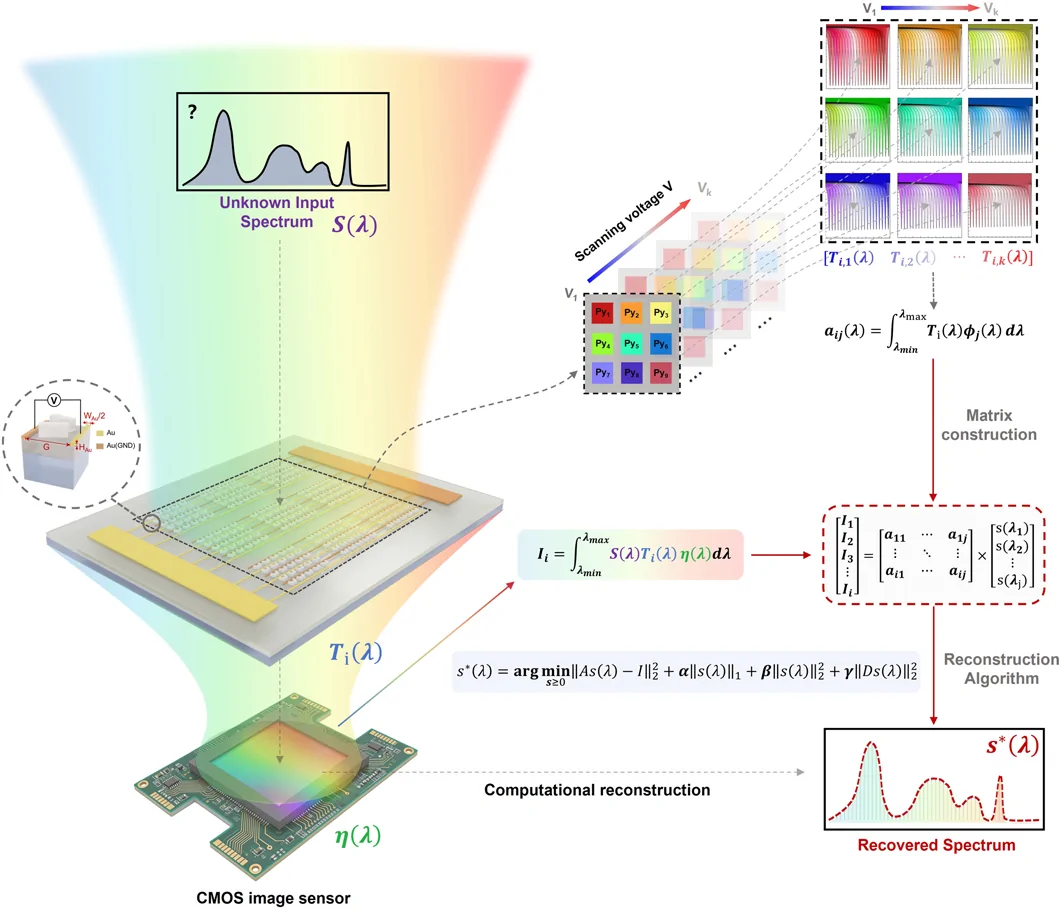
Schematic diagram of the miniaturized spectrometer based on the electrically tunable BTO metasurface.

The 3 × 3 BTO metasurface array delivers 89.47% optical throughput—defined as transmitted over incident flux—validating the high efficiency of the miniaturized spectrometer. We then computationally reconstruct spectra of various types: a single narrowband peak, two closely spaced narrow peaks, a continuous broadband spectrum, and a mixed spectrum with both broad and narrow features (see results in the Figure 6a–d). The reference spectra (gray solid lines) and algorithm‐reconstructed spectra (red dashed lines) are compared. The reconstruction fidelity is quantitatively evaluated using the metric defined in ref. [51],

(11) $$\text{Fidelity}=\frac{\sum_{i=1}^{j}\left(x_{i}\cdot {\tilde{x}}_{i}\right)}{\sqrt{\sum_{i=1}^{j}x_{i}^{2}\sum_{i=1}^{j}{\tilde{x}}_{i}^{2}}},$$

where $x_{i}$ is the *i*th data point of the original spectral signal and ${\tilde{x}}_{i}$ is the *i*th data point of the reconstructed spectral signal. The reconstruction in Figure 6a–d attain 99.11%, 99.30%, 99.99%, and 99.99% fidelity, respectively. Collectively yielding a remarkable average fidelity > 99%. Peaks separated by only 0.2 nm are cleanly discriminated (Figure 6b), validating the outstanding sub‐nanometers resolving power of the proposed miniaturized BTO‐metasurface spectrometer. Additional analysis of the spectral resolution for the two closely spaced narrowband peaks is provided in Supporting Information S1: Note 8.

**FIGURE 6:**
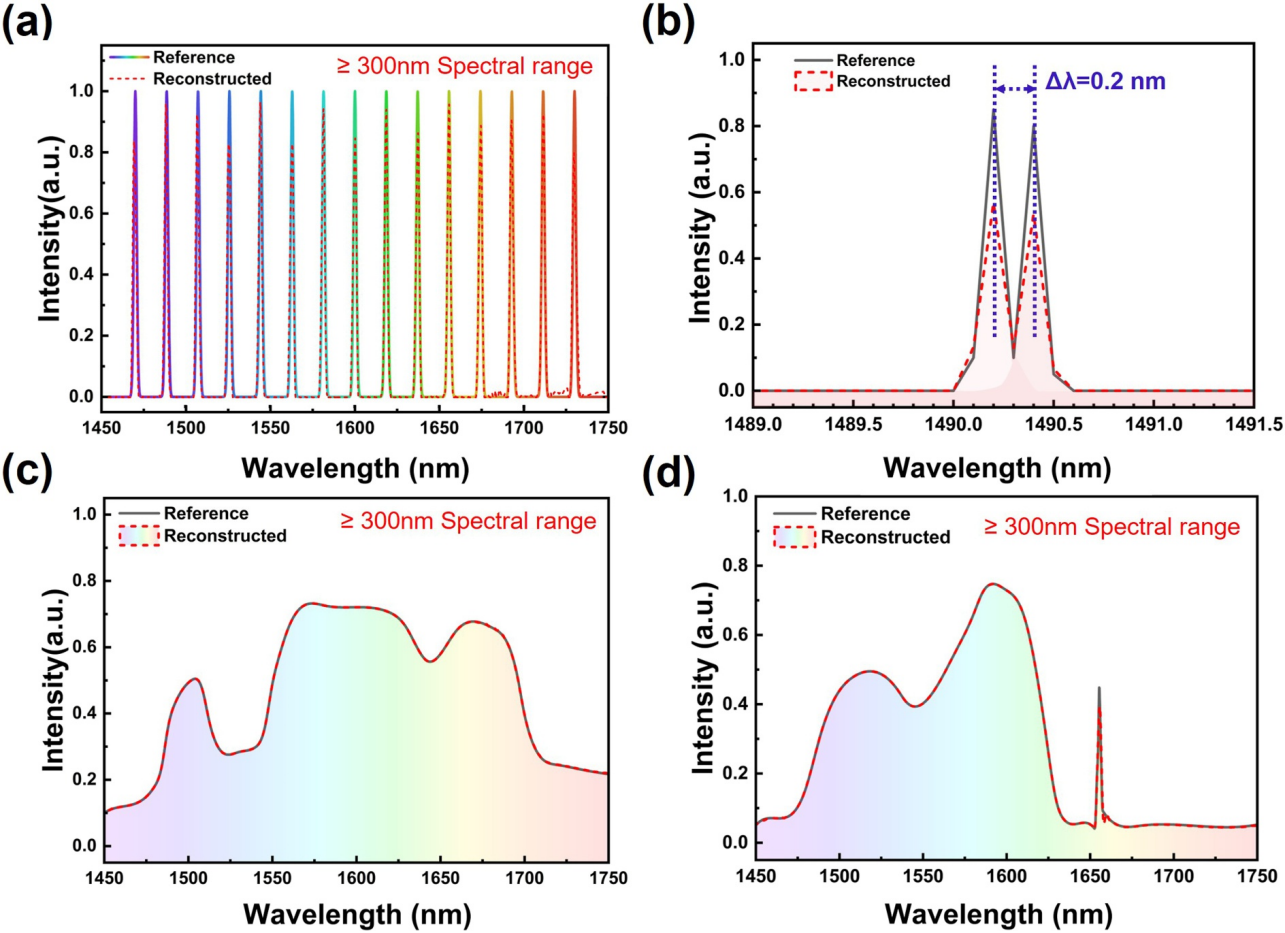
The BTO‐metasurface for high‐performance miniaturized spectrometer. Reconstructed spectrum: for narrowband single peak (a), two closely spaced narrowband peaks (resolution: 0.2 nm) (b), continuous broadband (c), and mixed broad and narrowband features (d).

Although primarily simulation‐based, this study includes a detailed analysis of the fabrication feasibility for the proposed electrically tunable metasurface. In Supporting Information S1: Note 9, we outline a preliminary fabrication process and discuss the impact of process‐induced errors. Alignment error analysis between the gold electrodes and a‐Si pillars demonstrates that the metasurface exhibits good robustness to such misalignment. Additionally, by deliberately avoiding etching of the BaTiO_3_ film and leveraging mature a‐Si etching techniques, sidewall effects are expected to be minimal. In Supporting Information S1: Note 10, we evaluate the converse piezoelectric deformation and Joule heating distribution under applied voltages. The results confirm that both effects are negligible within the operational electric field range, ensuring device stability. These feasibility analyses contribute to the practical application of ferroelectric thin films in tunable metasurfaces.

## 4 Conclusion

In summary, we demonstrate an active electrically tunable metasurface that hosts a quasi‐BIC (Q‐BIC) modes in BTO electro‐optic crystals, which enables static resonance wavelength tuning via geometric parameters and dynamic modulation through applied bias voltage, achieving an electrical tuning sensitivity of 1.79 nm/V. The supported Q‐BIC mode exhibits band‐stop filtering characteristics with narrow‐linewidth resonances, maintaining a high spectral resolution of 0.2 nm while achieving an average optical throughput of 89.47%. By integrating this metasurface into a miniaturized spectrometer and combining it with a spectral reconstruction algorithm, high‐accuracy spectral reconstruction is realized using only a 3 × 3 filter array and ∼20 measurement states (−10–10 V), with an average fidelity exceeding 99% and a core device footprint of only 250 μm^2^ total. The study thereby provides a viable platform for on‐chip spectral analysis and advances the use of BTO as a CMOS‐compatible route to reconfigurable high‐performance metasurface‐based metadevices.

## Author Contributions

**Jingyi Xia:** conceptualization, investigation, writing – original draft, writing – review and editing, visualization, methodology, formal analysis, data curation, software, validation. **Jietao Liu:** conceptualization, supervison, resources, visualization, project administration, writing – review and editing. **Yi Liu:** investigation, visualization, formal analysis. **Zhi Liu:** investigation, funding acquisition, supervision, resources, project administration. **Xin Wei:** resources, project administration, supervision, funding acquisition. **Guofeng Song:** funding acquisition, project administration, formal analysis, resources, supervision.

## Supporting information


Supporting Information S1

## Data Availability

The data that support the findings of this study are available upon reasonable request from the authors.
