# Supplementary material for: Electrically Reconfigurable Quasi‐BIC Metadevices Enabled by Integrated Barium Titanate
Source: Nanophotonics. 2026 Apr 25;15(9):e70104. doi: 10.1002/nap2.70104 (PMC13642558; doi:10.1002/nap2.70104)
Supplement: Supplementary file 1 [file NAP2-15-e70104-s001.docx]

**Supplementary Information for**

**Electrically Reconfigurable Quasi-BIC Metadevices Enabled by
Integrated Barium Titanate**

Jingyi Xia^1,4^,Jietao Liu^2^*,Yi Liu^1,4^ ,Zhi Liu^1,3,4^ **,Xin Wei^1,4^**,and Guofeng Song^1,4^

^1^ Institute of Semiconductors, Chinese Academy of Sciences, Beijing 100083, China.

^2^ Institute of Intelligent Photonics, Nankai University, Tianjin, 300071, China.

^3^ State Key Laboratory of Optoelectronic Materials and Devices, Institute of Semiconductors, Chinese Academy of Sciences, Beijing 100083, China

^4^ College of Materials Science and Opto-Electronic Technology, University of Chinese Academy of Sciences, Beijing 100049, China.

*Corresponding author: *liujietao@nankai.edu.cn*;

**Corresponding author: *zhiliu@semi.ac.cn; weix@semi.ac.cn.*

**Supplementary Note 1: Experimentally determined material parameters and simulation**

**Supplementary Note 2: Analysis of photon band structure**

**Supplementary Note 3: On the Transmission Spectral Response and Analysis of the Metasurface under TM Mode**

**Supplementary Note 4: CMT for EIT transmission spectrum**

**Supplementary Note 5: The calculation for group time delay**

**Supplementary Note 6: Analysis of electric‑optical overlap factor *Γ* and optimized BTO electro-optic coefficient through angle *φ***

**Supplementary Note 7: Optical Response of the Metasurface with Gold Electrodes**

**Supplementary Note 8: Reconstructed spectrum: for two closely spaced narrowband peaks**

**Supplementary Note 9: Fabrication Process and Tolerance Analysis of the Metasurface**

**Supplementary Note 10: Analysis of the Effects of Applied Voltage on Mechanical Strain and Joule Heating of the Metasurface**

**Supplementary Note 1: Experimentally determined material parameters and simulation**

We utilize experimentally determined optical constants for all materials used in this work. The refractive index and extinction coefficient of silicon are adopted from the experimental data compilation for Si in *Palik's* handbook ^[S1]^. The data for the barium titanate (BaTiO₃, BTO) and magnesium oxide (MgO) are taken from the curated experimental results available in the refractive index database (<https://refractiveindex.info/>) ^[S2]^, with the relationship between the refractive index and extinction coefficient of barium titanate as a function of wavelength shown in **Figure S1**. The optical constants for gold are sourced from the classic experimental measurements by *Johnso* and *Christy* ^[S3]^.

**Figure S1. Relationship between refractive index and extinction coefficient of barium titanate and wavelength (the data are interpolated and extrapolated to 2000 nm).**

Eigenmode analysis and electromagnetic field distributions are computed using the commercial finite element method (FEM) software COMSOL Multiphysics. Transmission spectra are simulated in parallel using the finite-difference time-domain (FDTD) method. The simulation domain employs Floquet periodic boundary conditions (PBC) along the *x* and *y* directions, with a perfectly matched layer (PML) implemented in the *z* direction to absorb outgoing radiation. A uniform mesh with a resolution of *Δx* = *Δy* = *Δz* = 10 nm is applied throughout the domain to ensure numerical accuracy.

**Supplementary Note 2: Analysis of photon band structure**

To investigate the modes of the designed metasurface in detail, we calculated its photonic band structure at orientation angle of *θ* = 0°, where the unit cell possesses *C₂* symmetry in the *X-Y* plane. Within the wavelength range of interest, four TE modes were identified, as shown in **Figure S2(a)**. We then examined the dependence of the Q‑factor and resonant wavelength at the *Γ* point for these four TE modes on *θ*. At *θ* = 0°, the Q‑factors of the TE_1_ and TE_2_ modes both approach ~10⁷ (effectively infinite; the calculated value is limited by the simulation mesh accuracy) and increase rapidly as *θ* decreases, confirming them as symmetry-protected bound states in the continuum (Symmetry-protected BIC). These correspond to the Q‑BIC_1_ and Q‑BIC_2_ modes in Figure 2 of the main text. In contrast, the TE_3_ and TE_4_ modes exhibit finite Q‑factors, corresponding to the GMR_1_ and GMR_2_ modes in Figure 2. As shown in the **Figure S2(b)**, the resonant wavelengths of the TE modes shift by less than 10 nm over the full range of *θ*, demonstrating the metasurface's robustness against fabrication variations.

**Figure S2. (a) Calculated photonic band structure. (b) Dependence of the TE mode's Q‑factor and resonance wavelength on orientation angle *θ*.**

**Supplementary Note 3: On the Transmission Spectral Response and Analysis of the Metasurface under TM Mode**

As shown in **Figure S2(a)**, within the near-infrared range of interest, the metasurface supports not only four TE modes (TE_1_, TE_2_, TE_3_, and TE_4_) but also three TM modes (indicated by the gray curves in **Figure S2(a)**). Corresponding to these three TM modes, we calculated the transmission spectra of the metasurface with the structural parameters of θ = 15° used in the main text under x-polarized incident light, as shown in **Figure S3(a)**. Three resonance peaks are clearly observed in the transmission spectra, with central wavelengths of 1460 nm, 1367 nm, and 1312 nm, corresponding to the TM_1_, TM_2_, and TM_3_ modes, respectively.

To analyze the nature of these three modes, we calculated their quality factors (Q-factors) in momentum space at θ = 0°, as shown in **Figure S3(c)**, and plotted the electric field component distributions at the Γ point, as displayed in the insets. The results indicate that TM_1_ and TM_3_ exhibit finite Q-factors (<10⁴), while TM_2_ shows an extremely high Q-factor exceeding 1×10⁷ at the Γ point. Furthermore, only the electric field distribution of the TM_2_ mode exhibits an odd symmetry, whereas those of TM_1_ and TM_3_ exhibit even symmetries. From these characteristics, it can be inferred that TM_2_ is a BIC mode transformed from a GMR, while TM_1_ and TM_3_ are GMR modes.

Subsequently, we performed a multipole decomposition analysis of the three TM modes, as presented in **Figure S3(b)**. Based on the scattering intensity of each multipole, both TM_1_ and TM_2_ are dominated by toroidal dipole (TD) contributions. The toroidal dipole is a special type of fundamental current excitation, as illustrated in **Figure S3(d)**. It manifests as closed loops formed by electric displacement vector arrows with opposite directions in the X-Y plane, resulting in two oppositely oriented magnetic field vectors. These loops close along the green arrows in the figure, forming toroidal dipoles oriented along the x-direction for TM_1_ (TD-x) and along the z-direction for TM_2_ (TD-z), respectively. For the TM_3_ mode, it is dominated by the electric quadrupole (EQ), with its four charges distributed at the interfaces between a-Si and BTO layer beneath the ends of the a-Si nanobars and at the interface between the BTO layer and the MgO layer.

**Figure S3. Optical response characteristics of the metasurface in TM mode.** (a) Transmission spectra, (b) Multipole decomposition results of the scattering cross-section at the corresponding resonant positions, (c) Electric field distribution E and magnetic field distribution H at the three resonant positions. The white arrows indicate the in-plane electric field vector direction, the "±" symbols mark the centers of positive and negative bound charges forming the electric dipole moment, and the green arrows indicate the closed loop direction of the magnetic dipole.

**Supplementary Note 4: CMT for EIT transmission spectrum**

To analyze the EIT phenomenon observed in the transmission spectrum due to the coupling between the GMR_1_ and Q-BIC_2_ modes, we utilize the coupled-mode theory (CMT) in the main text. The corresponding coupled-mode equations are as follows ^[S4, S5]^,

$$\left\{ \begin{aligned} \frac{\text{d}\text{a}_{\text{1}}}{\text{dt}}\text{=}\left( \text{j}\text{ω}_{\text{1}}\text{-}\text{γ}_{\text{1}} \right)\text{a}_{\text{1}}\text{+}\text{jκ}\text{a}_{\text{2}}\text{+}\sqrt{\text{γ}_{\text{1r}}}\text{s}_{\text{+}} \\ \frac{\text{d}\text{a}_{\text{2}}}{\text{dt}}\text{=}\left( \text{j}\text{ω}_{\text{2}}\text{-}\text{γ}_{\text{2}} \right)\text{a}_{\text{2}}\text{+}\text{jκ}\text{a}_{\text{1}} \end{aligned}\text{ }\text{ (1)} \right.$$

Here, $\text{a}_{\text{1}}$ and $\text{a}_{\text{2}}$ denote the mode amplitudes of GMR_1_ and Q-BIC_2_, respectively; $\text{ω}_{\text{1}}$ and $\text{ω}_{\text{2}}$ are their resonant frequencies; and $\text{γ}_{\text{1}}$ and $\text{γ}_{\text{2}}$ are the corresponding total loss rates, where $\text{γ}_{\text{i}}\text{=}\text{γ}_{\text{ir}}\text{+}\text{γ}_{\text{ia}}$ ( *i* denotes 1 or 2) . In these expressions, $\text{γ}_{\text{ir}}$ and $\text{γ}_{\text{ia}}$ represent radiative and absorptive loss, respectively, $\text{κ}$ is the coupling coefficient between the two modes, and $\text{s}_{\text{+}}$ is the amplitude of the incident field. Solving the coupled equations under steady-state conditions yields,

$$\left\{ \begin{aligned} \text{a}_{\text{2}}\text{=}\frac{\text{jκ}}{\text{j}\left( \text{ω-}\text{ω}_{\text{2}} \right)\text{+}\text{γ}_{\text{2}}}\text{a}_{\text{1}} \\ \text{a}_{\text{1}}\text{=}\frac{\sqrt{\text{γ}_{\text{1r}}}}{\text{j}\left( \text{ω-}\text{ω}_{\text{1}} \right)\text{+}\text{γ}_{\text{1}}\text{+}\frac{\text{κ}^{\text{2}}}{\text{j}\left( \text{ω-}\text{ω}_{\text{2}} \right)\text{+}\text{γ}_{\text{2}}}}\text{s}_{\text{+}} \end{aligned}\text{ }\text{ (2)} \right.$$

Under transmission conditions, the output field $\text{s}_{\text{-}}$ and the corresponding transmission coefficient $\text{t}\left( \text{ω} \right)$ can be expressed as,

$$\left\{ \begin{aligned} \text{s}_{\text{-}}\text{=}\text{s}_{\text{+}}\text{-}\sqrt{\text{γ}_{\text{1r}}}\text{a}_{\text{1}} \\ \text{t}\left( \text{ω} \right)\text{=}\frac{\text{s}_{\text{-}}}{\text{s}_{\text{+}}}\text{=1-}\frac{\text{γ}_{\text{1r}}}{\text{j}\left( \text{ω-}\text{ω}_{\text{1}} \right)\text{+}\text{γ}_{\text{1}}\text{+}\frac{\text{κ}^{\text{2}}}{\text{j}\left( \text{ω-}\text{ω}_{\text{2}} \right)\text{+}\text{γ}_{\text{2}}}} \end{aligned}\text{ }\text{ }\text{ }\text{ }\text{ }\text{ (3)} \right.$$

This leads to the transmission equation used for CMT fitting of the EIT spectrum in the main text (using the relation $\text{ω=}\text{2}\text{πc/λ}$),

$$\text{ }\text{T}\left( \text{λ} \right)\text{=}\left| \text{t}\left( \text{ω} \right) \right|^{\text{2}}\text{=}\left| \text{1-}\frac{\text{γ}_{\text{1r}}}{\text{j·2}\text{π}\text{c}\left( \frac{\text{1}}{\text{λ}}\text{-}\frac{\text{1}}{\text{λ}_{\text{1}}} \right)\text{+}\text{γ}_{\text{1}}\text{+}\frac{\text{κ}^{\text{2}}}{\text{j·2}\text{π}\text{c}\left( \frac{\text{1}}{\text{λ}}\text{-}\frac{\text{1}}{\text{λ}_{\text{2}}} \right)\text{+}\text{γ}_{\text{2}}}} \right|^{\text{2}}\text{ }\text{ }\text{ }\text{ }\text{(4)}$$

Here, $\text{λ}_{\text{1}}$ and $\text{λ}_{\text{2}}$ denote the resonant wavelengths corresponding to the GMR_1_ and Q-BIC_2_ modes.

**Supplementary Note 5: The calculation for group time delay**

The slow light effect is generally characterized by the group delay (T_g_), defined as T_g_=-d*φ*/d*ω*, where *φ* is the transmission phase delay and *ω* is the angular frequency of the incident light. As shown in **Figure S4**, we calculated the transmission phase and the corresponding group delay of the metasurface. It can be observed that the ultra-sharp EIT transparency window, resulting from the strong coupling interaction between the GMR_1_ mode and the Q-BIC_2_ mode, has a resonance center at 1383.7 nm. At this wavelength, a group delay of T_g_ = 11.4 ps is achieved, which is the maximum group delay among the four modes. At the center of this extremely narrow transparency window, due to destructive interference, radiation loss is significantly suppressed, and energy is confined within the structure for an extended period. The sharp change in transmittance (amplitude) is inevitably accompanied by a drastic variation in phase. Specifically, at the center of this transparency window, the rate of change of phase with respect to frequency (d*φ*/d*ω*) reaches its maximum negative value, thereby generating the maximum positive group delay (11.4 ps).

In comparison, at 1371.7 nm, the GMR_1_ mode achieves a group delay of 6.3 ps, while at 1488.2 nm, the Q-BIC_1_ mode exhibits a group delay of -15.5 ps, manifesting as strong anomalous dispersion. For the TM_3_ mode at 1312 nm, its group delay is negligible. This is because this wavelength is far from any high-Q resonance center, or the coupling between the TM_3_ mode and free space is extremely weak, resulting in a nearly linear and slow variation of phase with frequency, thus leading to an almost zero group delay. The TM_2_ mode at 1367 nm exhibits a very strong anomalous dispersion, with a group delay of -77.3 ps. Near the TM_1_ mode around 1460 nm, the group delay rapidly decreases from 6.3 ps at 1460.80 nm to -82.8 ps at 1460.86 nm, demonstrating a dramatic reversal from positive to negative within a wavelength range of less than 0.06 nm. At 1460.80 nm, the system is on the "transparent" or "low-loss" side of the resonance, characterized by energy storage; whereas at 1460.86 nm, it crosses the resonance center and enters the anomalous dispersion region. Such a sharp transition in group delay suggests the presence of a transmission amplitude minimum, or even a zero point, in this vicinity. Near the amplitude minimum, the phase undergoes an abrupt change on the order of φ. The derivative of this abrupt phase change in the frequency domain (d*φ*/d*ω*) forms an extremely narrow and high peak (whether positive or negative). The observed group delay of -82.8 ps is precisely the manifestation of this phase singularity in the frequency domain.

**Figure S4 Transmission spectra, transmission phase, and group delay.** (a) Transmission spectra featuring GMR_2_, GMR_1_, Q-BIC_2_, and EIT, (b) Transmission phase corresponding to panel (a), (c) Group delay corresponding to panel (a), (d) Transmission spectra of Q-BIC_1_, (e) Transmission phase corresponding to panel (d), (f) Group delay corresponding to panel (d), (g) Transmission spectra of TM_1_, TM_2_, and TM_3_, (h) Transmission phase corresponding to panel (g), (i) Group delay corresponding to panel (g).

**Supplementary Note 6: Analysis of electric‑optical overlap factor *Γ* and optimized BTO electro-optic coefficient through angle** $\text{φ}$

**4.1 Analysis of the electric‑optical overlap factor *Γ***

The structure discussed in this work supports a quasi-bound state (Q-BIC_1_) in the continuum for electrically tunable functionality. To achieve efficient electro‑optic conversion, strong overlap between the optical near‑field of the resonator and the DC electric field applied across the BTO electro‑optic material is required, as well as alignment of their vector directions as parallel as possible. In **Figure S5**, we present simulated cross‑sections of both the optical and electric field distributions.

From cross‑sections A, B, and C, it is observed that the *TE₁* mode corresponding to Q-BIC_1_ is excited by *y*‑polarized light, while its near‑field distribution in the resonator is primarily *x*‑polarized. By aligning the electrodes parallel to the *y*‑axis, the electric field vector is made parallel to the optical field vector, thereby ensuring efficient electro‑optic operation. Furthermore, cross‑section D reveals that due to the strong localization of the Q‑BIC_1_ mode, the optical field is highly confined at both ends of the *a‑Si* rod and within the BTO layer. This results in pronounced overlap between the optical field and the BTO electro‑optic material, creating favorable conditions for high‑efficiency electro‑optic conversion.

The electric‑optical overlap factor, denoted as *Γ*, is one of the key parameters in the Pockels effect. Since the applied electric field is not always uniformly distributed within the optical mode region, *Γ* quantifies the spatial matching between the external electric field and the optical mode field. It essentially characterizes the extent to which the applied electric field effectively interacts with the optical field. The expressions for $\text{Γ}$ used in this work are given as,

$$\text{ }\text{Γ}\text{=}\frac{\text{1}}{\text{E}_{\text{A}}}\text{ }\frac{\int_{\text{BTO}} \text{E}_{\text{e}}\left| \text{E}_{\text{op}} \right|^{\text{2}}\text{d}\text{V}_{\text{BTO}}}{\int_{\text{BTO}} \left| \text{E}_{\text{op}} \right|^{\text{2}}\text{d}\text{V}_{\text{BTO}}}\text{ }\text{ }\text{ (5)}$$

From cross-section D of **Figure S5**, it can be observed that the electric field within the BTO layer exhibits a non-uniform distribution. To evaluate $\text{Γ}$ more rigorously, we introduce the electric field per unit volume, $\text{E}_{\text{A}}$,

$${\text{ }\text{E}}_{\text{A}}\text{= }\frac{\int_{\text{BTO}} \left| \text{E}_{\text{V}} \right|\text{d}\text{V}_{\text{BTO}}}{\text{V}_{\text{BTO}}}\text{ (6)}$$

with an applied bias voltage of 10 V, simulations yield an average electric field of $\text{E}_{\text{A}}\text{ }\text{≈}\text{ }\text{8.21×}\text{10}^{\text{6}}\text{ }\text{V}\text{/m}$, leading to a electric–optical overlap factor $\text{Γ}\text{ }\text{≈}\text{ }\text{0.8}$.

**Figure S5. Optical and electro-static simulations.** **The distributions of the electric field component *Ex* and the absolute value of the electric field component *|Ex|* for optical and DC fields corresponding to the Q-BIC_1_ resonance peak at different cross-sections (A, B, C, D), along with the electric displacement vectors (indicated by white arrows).**

**4.2 Optimized BTO electro-optic coefficient through angle *φ***

The barium titanate (BTO) electro-optic crystal has three main electro-optic coefficients, $\text{r}_{\text{1}\text{3}}=\text{r}_{\text{2}\text{3}}$, $\text{r}_{\text{3}\text{3}}$, and $\text{r}_{\text{42}}=\text{r}_{\text{51}}$. According to the crystal symmetry, its electro-optic tensor can be written in the following form ^[S6]^,

$$\text{r}_{\text{BTO}}\text{=}\left( \begin{matrix} \begin{matrix} \text{0} \\ \text{0} \\ \text{0} \end{matrix} & \begin{matrix} \text{0} \\ \text{0} \\ \text{0} \end{matrix} & \begin{matrix} \text{r}_{\text{13}} \\ \text{r}_{\text{23}} \\ \text{r}_{\text{33}} \end{matrix} \\ \begin{matrix} \text{0} \\ \text{r}_{\text{51}} \\ \text{0} \end{matrix} & \begin{matrix} \text{r}_{\text{42}} \\ \text{0} \\ \text{0} \end{matrix} & \begin{matrix} \text{0} \\ \text{0} \\ \text{0} \end{matrix} \end{matrix} \right)\text{ }\text{(}\text{7}\text{)}$$

According to the theoretical study on BTO electro-optic modulation by *P. Castera* et al. ^[S7]^, the relationship between the effective electro-optic coefficient${\text{ }\text{r}}_{\text{eff}}$ and $\text{φ}$ (the angle between the optical axis of the BTO crystal and the applied electric field $\text{E}$), as well as the electro-optic coefficients, is expressed by the following formula,

$${\text{ }\text{r}}_{\text{eff}}\left( \text{φ} \right)\text{ }\text{=}{\text{ }\text{r}}_{\text{33}}\text{cos}^{\text{3}}\left( \text{φ} \right)\text{+}\left( \text{r}_{\text{13}}\text{+2}\text{r}_{\text{51}} \right)\text{sin }^{\text{2}}\left( \text{φ} \right)\text{cos}\left( \text{φ} \right)\text{ (}\text{8}\text{)}$$

Based on the parameters from the main text, the relationship between the effective electro-optic coefficient and the angle $\text{φ}$ (between the applied electric field and the optical axis of the BTO crystal) is calculated, as shown in **Figure S6**. The result indicates that $\text{r}_{\text{eff}}$ equals $\text{r}_{\text{3}\text{3}}$ when $\text{φ}$ is 0° (or 180°), while the maximum effective electro-optic coefficient, $\text{r}_{\text{eff}\text{,}\text{max}}\text{=}\text{791 pm/V}$, is achieved at $\text{φ}$ = 50°.

**Figure S6. Dependence of the effective electro-optic coefficient on the angle** $\text{φ}$ **(between the applied field and the BTO optical axis).**

**Supplementary Note 7: Optical Response of the Metasurface with Gold Electrodes**

To provide a clearer illustration of the optical response of the metasurface upon the introduction of gold electrodes, we have systematically reorganized and supplemented the relevant analysis, as detailed below.As shown in **Figure S7**, we calculated the transmission spectra for three different metasurface configurations: the metasurface with only amorphous silicon (a-Si) nanobar pairs, the structure with only gold electrodes (without a-Si pairs), and the structure integrating both gold electrodes and a-Si pairs. For the structure with only a-Si pairs, its spectral characteristics have been thoroughly discussed in the main text, exhibiting four resonance peaks in the near-infrared region, corresponding to the GMR_2_, GMR_1_, Q-BIC_2_, and Q-BIC_1_ modes, respectively. The structure containing only gold electrodes is essentially equivalent to a one-dimensional rectangular metallic grating. Its transmission spectrum displays a broad resonance peak centered at 1346.8 nm with a full width at half maximum (FWHM) of approximately 16 nm. Due to the intrinsic Ohmic losses of gold, the background transmittance of this resonance peak is lower than that of the all-dielectric metasurface.

**Figure S7. Comparison of transmission spectra for different BTO metasurface structures. From left to right: transmission spectra of metasurfaces consisting solely of amorphous silicon pillar pairs on the MgO/BTO layer, those with only gold interdigital electrodes, and those with both gold interdigital electrodes and amorphous silicon rod pairs, respectively. The red triangles mark the central wavelengths of the resonance peaks dominated by the gold electrodes, while the blue circles mark the central wavelength of the Q-BIC1 resonance peak.**

When both the gold electrodes and a-Si pairs are integrated, the resonance peak dominated by the gold electrodes is preserved. However, the resonance peaks corresponding to GMR_2_, GMR_1_, and Q-BIC_2_, which are spectrally close to this mode, completely disappear from the transmission spectrum. In contrast, the Q-BIC_1_ mode, whose resonance frequency is spectrally distant from the gold electrode resonance, is less affected. It only exhibits a minor redshift (the central wavelength shifting from 1488.2 nm to 1492.5 nm) and a slight increase in transmittance at the central wavelength (from near zero to 0.045). To further elucidate the physical mechanism behind this phenomenon, we calculated the electric field distributions (X-Y, Y-Z, and X-Z cross-sections) at the two resonance peaks (marked by the red triangle and blue circle in **Figure S7**) for the structure integrating both electrodes and a-Si pairs. The results are presented in **Figure S8**.

**Figure S8. Electric field distribution E at the two resonance peaks of the metasurface structure simultaneously arranged with y-direction gold interdigital electrodes and amorphous silicon pillar pairs.** (a) Electric field distribution in the X-Y plane at 1346.8 nm, (b) Electric field distribution in the Y-Z plane at 1346.8 nm, (c) Electric field distribution in the X-Z plane at 1346.8 nm, (d) Electric field distribution in the X-Y plane at 1492.5 nm, (e) Electric field distribution in the Y-Z plane at 1492.5 nm, (f) Electric field distribution in the X-Z plane at 1492.5 nm.

Given that only the Q-BIC_1_ mode retains a relatively high quality factor (Q-factor) and extinction ratio after the introduction of the gold electrodes, the subsequent analysis primarily focuses on the impact of the electrodes on this mode. As shown in **Figure S8(d)**, the introduction of the gold electrodes induces new resonant coupling between the a-Si pairs and the electrodes. This causes a portion of the energy of the Q-BIC_1_ mode to be absorbed and dissipated by the gold electrodes, consequently leading to a reduction in the Q-factor and a shift in the resonance wavelength.

We further investigated the influence of the structural parameters of the gold electrodes—namely, the height *H_Au_* and width *W_Au_*—on the Q-BIC_1_ resonance. The results are presented in **Figure S9**. As depicted in **Figure S9(a)** and **(b)**, upon the introduction of the gold electrodes, the central wavelength of the Q-BIC_1_ resonance undergoes a significant redshift (from 1488 nm to approximately 1492 nm), and the Q-factor decreases from 2128 to 1213. As the electrode height *H_Au_* increases from 20 nm to 90 nm, the central wavelength exhibits a slight blueshift of about 1 nm, the Q-factor further decreases to 937, and the transmittance at the central wavelength shows a minor increase. These simulation results indicate that the height of the gold electrodes has a relatively minor effect on the Q-BIC_1_ resonance.

In comparison, the width of the gold electrodes *W_Au_* has a more pronounced impact on the Q-BIC_1_ resonance. As shown in **Figure S9(c)** and **(d)**, when *W_Au_* increases from 40 nm to 180 nm, the resonance peak undergoes a significant redshift. Concurrently, the Q-factor drops sharply from 2128 to 286, and the transmittance at the central wavelength rises from 0.0019 to 0.4673. To interpret this phenomenon, we calculated the electric field distributions in the X-Y plane at the central wavelength for different electrode widths, as presented in **Figure S10**. As the electrode width increases, the electric field originally localized at the ends of the a-Si nanobars gradually diminishes and transfers to the vicinity of the gold electrodes. This transfer results in substantially enhanced absorption loss of the mode's energy by the gold electrodes. Therefore, to maintain a high-Q resonance, the width of the gold electrodes should be confined to within 80 nm.

**Figure S9. Influence of the gold electrode structure on the transmission characteristics.** (a) Effect of the gold electrode height *H_Au_* on the Q-BIC_1_ resonance peak, (b) Effect of the gold electrode height *H_Au_* on the Q-factor and transmittance at the central wavelength *λ_0_* of the Q-BIC_1_ mode, (c) Effect of the gold electrode width *W_Au_* on the Q-BIC_1_ resonance peak, (d) Effect of the gold electrode width *W_Au_* on the Q-factor and transmittance at the central wavelength *W_Au_* of the Q-BIC_1_ mode.

**Figure S10. Electric field distribution in the X-Y cross-section at the central wavelength for different gold electrode widths.**

In summary, the introduction of gold electrodes primarily affects the Q-BIC1 mode by reducing its Q-factor and shifting its resonance wavelength. The electrode width is identified as the dominant factor influencing these changes, while the electrode height plays a comparatively minor role. These analyses provide crucial guidance for the design of electrically tunable metasurfaces.

**Supplementary Note 8: Reconstructed spectrum: for two closely spaced narrowband peaks**

The ability to resolve narrowly spaced spectral peaks is a crucial function of miniature spectrometers. In the main text, we demonstrated that the designed microspectrometer achieves a best achievable resolution of 0.2 nm. Here, we further supplement the spectral reconstruction results for two closely spaced narrowband peaks with different wavelength separations to illustrate the device’s spectral resolution capability, as shown in **Figure S11**. The metadevice can clearly distinguish two narrow peaks separated by 0.3 nm, 0.4 nm, 0.5 nm, and 1 nm, demonstrating excellent spectral resolution power.

**Figure S11. Reconstructed spectrum: for two closely spaced narrowband peaks.** (a) resolution: 0.3 nm,(b) resolution: 0.4 nm,(c) resolution: 0.5 nm,(d) resolution: 1.0 nm.

**Supplementary Note 9: Fabrication Process and Tolerance Analysis of the Metasurface**

Regarding the fabrication pathway, we have provided a preliminary outline in the manuscript, as illustrated in the newly added **Figure S12**. This figure schematically depicts the proposed process flow for the proposed barium titanate-based electrically tunable metasurface. The procedure begins with organic cleaning of a single-side polished MgO substrate to ensure surface cleanliness. Subsequently, a Ni₈₀Fe₂₀ permalloy layer is deposited onto the backside of the MgO substrate via magnetron sputtering to enhance thermal conductivity. Following this, an a-axis-oriented BTO single-crystal thin film is epitaxially grown on the front side of the MgO substrate using molecular beam epitaxy. After the BTO film growth, chemical mechanical polishing is employed to remove the permalloy layer from the backside, followed by thinning and polishing of the MgO substrate backside to improve optical transparency. Next, an a-Si thin film and a SiO₂ hard mask are sequentially deposited via plasma-enhanced chemical vapor deposition. After photoresist spinning, the pattern is first transferred to the photoresist layer via photolithography and then to the SiO₂ layer through reactive ion etching. Using the SiO₂ layer as a hard mask, the pattern is further defined into the a-Si layer by inductively coupled plasma etching, after which the residual SiO₂ is removed using a buffered oxide etch solution. Finally, gold interdigitated electrodes are fabricated via a bilayer lift-off process: a layer of LOR and a positive photoresist are sequentially spin-coated, followed by exposure and development to form an undercut profile. Metal deposition is then carried out by magnetron sputtering, and the electrodes are obtained after the lift-off procedure.

**Figure S12. Schematic illustration of the fabrication process for the barium-titanate-based electrically tunable metasurface.**

With respect to tolerance analysis, alignment error primarily arises from the overlay accuracy between the gold interdigitated electrodes and the a-Si pillars. As indicated in **Figure S13(b)**, during fabrication, a relative positional shift along the x-direction may occur between the gold electrodes and the a-Si bars, and we denote this misalignment using the parameter dx. The influence of this offset on the Q-BIC₁ resonance is evaluated in **Figure S14**. Simulation results reveal that the alignment error dx has virtually no effect on the full width at half maximum of the Q-BIC₁ resonance. As dx increases from 0 nm to 80 nm, the resonance peak exhibits a redshift of approximately 0.5 nm, accompanied by a slight increase in transmittance at the central wavelength from 0.048 to 0.075. These findings indicate that the proposed metasurface device with gold interdigitated electrodes is largely insensitive to alignment inaccuracies, thereby demonstrating favorable process robustness.

**Figure S13. Schematic illustration of the electrically tunable BTO metasurface structure after introducing gold interdigital electrodes.** (a) Structural schematic of the BTO metasurface array and a unit cell, (b) X-Y plane top view of the BTO metasurface unit cell, (c) X-Z plane side view of the BTO metasurface unit cell.

**Figure S14. Effect of the positional shift dx of the gold electrode along the x-direction relative to the a-Si rod on the Q-BIC_1_ resonance peak.**

Concerning the sidewall profile, previous experimental studies have shown that etching of barium titanate electro-optic crystals often inevitably results in slanted sidewalls. For instance, Stefan Abel et al.[S8] observed pronounced sidewall inclination even when etching only ~40 nm of barium titanate during the fabrication of plasmonic waveguides via ion beam etching. In light of this, we have taken such considerations into account during the initial design phase of the metasurface structure. In our configuration, barium titanate serves as the intermediate layer responsible for the electro-optic response and does not require any etching or patterning, thereby circumventing the issue of sidewall slope altogether. The only etching step involves the topmost a-Si bars with a height of 180 nm, for which silicon etching—particularly shallow etching—has already reached a high level of maturity. For example, Stefan Abel et al. [S8] fabricated silicon waveguides on barium titanate thin films using inductively coupled plasma etching, achieving highly vertical sidewalls. Moreover, Chenqian Wang et al. [S9] reported silicon nanostructures in a metasurface configuration with dimensions comparable to those of our a-Si bars; they also obtained exceptionally vertical sidewall profiles via inductively coupled plasma etching. In summary, we are confident that the vertical sidewall etching of the silicon nanostructures proposed in our design can be reliably realized using existing well-established fabrication techniques.

**Supplementary Note 10: Analysis of the Effects of Applied Voltage on Mechanical Strain and Joule Heating of the Metasurface**

We would like to provide further elaboration regarding the piezoelectric characteristics of barium titanate (BaTiO_3_) and its potential influence on the electrically tunable metasurface. Barium titanate is a quintessential ferroelectric material and stands as one of the earliest discovered and most widely utilized piezoelectric materials. At room temperature, BaTiO_3_ crystallizes in a tetragonal structure that lacks a center of symmetry, which is a necessary condition for the occurrence of piezoelectricity. When an external force is applied to the crystal, lattice deformation occurs, leading to relative ionic displacements that alter the spontaneous polarization—this is known as the direct piezoelectric effect. Conversely, the application of an external electric field modifies the polarization state and induces lattice deformation, corresponding to the converse piezoelectric effect. In the electrically tunable metasurface proposed in this study, the converse piezoelectric effect of BaTiO_3_ may influence device performance, thus necessitating a dedicated simulation analysis.

To investigate this effect, we employed the "Piezoelectric Devices" predefined multiphysics interface within the commercial finite element software COMSOL Multiphysics to simulate the converse piezoelectric response of BaTiO_3_ in the device. Material parameters were adopted directly from the "BaTiO_3_ [solid, pure]" data available in the COMSOL Material Library. The piezoelectric properties were configured based on the strain-charge constitutive relation. In this formulation, the compliance matrix s describes the linear relationship between stress and strain. For a tetragonal crystal system (point group 4mm), the compliance matrix s is a symmetric 6×6 matrix characterized by six independent constants: s_11_, s_12_, s_13_, s_33_, s_44_, and s_66_. These constants represent the axial elastic compliance (along the a-axis), transverse Poisson coupling, axial–transverse coupling, axial elastic compliance (along the c-axis/polar axis), shear compliance (about the a- or b-axis), and shear compliance (about the c-axis), respectively, with units in 1/TPa. These constants are arranged using Voigt notation, and the resulting matrix is expressed as follows:

$\text{s }\text{=}\left[ \begin{matrix} \begin{matrix} s_{11} & s_{12} & s_{13} \\ s_{12} & s_{11} & s_{13} \\ s_{13} & s_{13} & s_{33} \end{matrix} & \begin{matrix} 0 & 0 & 0 \\ 0 & 0 & 0 \\ 0 & 0 & 0 \end{matrix} \\ \begin{matrix} 0 & 0 & 0 \\ 0 & 0 & 0 \\ 0 & 0 & 0 \end{matrix} & \begin{matrix} s_{44} & 0 & 0 \\ 0 & s_{44} & 0 \\ 0 & 0 & s_{66} \end{matrix} \end{matrix} \right]\text{ }$	(9)

Similarly, in the strain-charge constitutive relation, the piezoelectric coupling matrix d links the electric field to the strain. For a tetragonal crystal system, the piezoelectric coupling matrix is a 3×6 matrix characterized by three independent constants: d_31_, d_33_, and d_15_, which correspond to the transverse, longitudinal, and shear piezoelectric coefficients, respectively, with units in pC/N. These constants are arranged using Voigt notation, and the resulting matrix is expressed as follows:

$\text{d }\text{=}\left[ \begin{matrix} \begin{matrix} 0 & 0 & 0 \\ 0 & 0 & 0 \\ d_{31} & d_{31} & d_{33} \end{matrix} & \begin{matrix} 0 & d_{15} & 0 \\ d_{15} & 0 & 0 \\ 0 & 0 & 0 \end{matrix} \end{matrix} \right]\text{ }$	(10)

We appreciate the reviewer's attention to the selection of simulation parameters. Due to the lack of direct experimental data on the piezoelectric properties of barium titanate thin films, our simulations referred to the parameters of bulk barium titanate crystals reported by M. Zgonik et al. [S10], in which d_33_ was taken as 90 pC/N. It is worth noting that Yiping Guo et al. [S11] reported a significant thickness dependence of the piezoelectric coefficient in barium titanate films. For a barium titanate film with a thickness of approximately 280 nm, the measured d_33_ was about 40 pm/V (the units pm/V and pC/N are equivalent), which is considerably lower than the bulk reference value. Therefore, it can be anticipated that in actual devices, the deformation induced by the converse piezoelectric effect will be smaller than that predicted by the simulations presented in this work. In other words, the current simulations may somewhat overestimate the influence of the converse piezoelectric effect on the tuning performance of the metasurface.

Based on the aforementioned model, we simulated the deformation of the metasurface structure induced by the converse piezoelectric effect of barium titanate upon applying a voltage to the gold interdigitated electrodes, with the results presented in **Figure S15**. Alternating potentials of 10 V and 0 V were applied to the strip-shaped gold interdigitated electrodes. **Figure S15(a)** illustrates the schematic of the electrically tunable metasurface structure designed in this study, where the geometric parameters are consistent with those described in the main text. **Figures S15(b)–(d)** display the three-dimensional deformation distribution, the X-Z cross-sectional view, and the Y-Z cross-sectional view, respectively, resulting from the converse piezoelectric effect under an applied voltage of 10 V. To visualize the deformation more clearly, the displacement is scaled by a factor of 150. The simulation results indicate that the maximum deformation is only 0.206 nm under a driving voltage of 10 V. The surface of the barium titanate film exhibits alternating concave and convex patterns on either side of the gold electrodes, leading to corresponding undulations at the bottom of the a-Si pillars in adjacent metasurface unit cells. Considering that the piezoelectric coefficient d_33_ of actual barium titanate thin films is generally lower than the bulk value used in the simulation, the actual maximum deformation caused by the converse piezoelectric effect is expected to be even smaller than 0.206 nm. Compared with the minimum feature size of the metasurface (b = 120 nm), this deformation is negligible. Furthermore, we calculated the maximum deformation induced by the converse piezoelectric effect over a voltage range from 0 V to 50 V, as shown in **Figure S16**. The results demonstrate that even at an applied voltage of 50 V, the maximum deformation is only 1.03 nm, further confirming that the influence of the converse piezoelectric effect on the device performance is negligible.

**Figure S15. Deformation results of the electrically tunable metasurface under a voltage of 10 V due to the inverse piezoelectric effect of barium titanate.** (a) Schematic diagram of the electrically tunable metasurface, (b) 3D deformation result under 10 V, (c) X-Z cross-sectional view, (d) Y-Z cross-sectional view.

**Figure S16. Effect of inverse piezoelectric deformation under different voltages.**

We further discuss the source of Joule heating in the electrically tunable metasurface device and its potential impact on device performance. In the proposed design, since the interdigitated electrodes are fabricated using gold with high electrical conductivity, the Joule heating generated by the electrodes themselves is negligible. Therefore, the primary source of Joule heating in the device is the leakage current originating from the barium titanate ferroelectric thin film. In our previous electro-optic simulations, to simplify the calculations, we treated the BaTiO_3_ film as an ideal insulator. However, in practice, structural defects such as oxygen vacancies and dislocations are inevitably introduced during film fabrication. These defects tend to form localized conductive paths, leading to leakage currents in the BaTiO_3_ film near the gold electrodes.

It has been reported that the leakage mechanism in BaTiO_3_ thin films is strongly dependent on the applied electric field. Under low electric fields (below 20 kV/cm), the current exhibits a linear relationship with voltage, and the leakage behavior typically follows an Ohmic conduction model. As the electric field increases, the conduction mechanism may transition. Under high electric fields, the leakage current behavior may be governed by the space-charge-limited current mechanism or the Schottky emission model. In the compact structure of the electrically tunable metasurface proposed in this study, high-field conditions are unavoidable. Taking an applied voltage of 10 V as an example, a preliminary estimation indicates that the electric field introduced by the gold interdigitated electrodes is approximately 140 kV/cm, which significantly exceeds the low-field threshold.

Wei Zhang et al. [S12] systematically investigated the leakage behavior of 300-nm-thick BaTiO_3_ films grown on MgO substrates under various electric fields. Their results demonstrate that within the electric field range of 40 kV/cm to over 660 kV/cm, the conduction mechanism of the BaTiO_3_ film conforms to the Schottky emission model. Based on this model, the leakage current density can be expressed as:

$\text{ln(J) }\text{= }\frac{q}{kT}\sqrt{\frac{q^{3}}{4\pi\varepsilon_{0}\varepsilon_{d}}}\sqrt{E}+\ln\left( AT^{2} \right)-\frac{q\phi_{B}}{kT}$	(11)

In the Schottky emission model, the effective Richardson constant *A* is taken as 120 A/(cm²·K²), as reported in Ref. [S13]. The Schottky barrier height ϕ_B_ and the high-frequency dielectric constant ε_d_ of BaTiO₃ are extracted from the experimental data reported by Wei Zhang et al. [S12]. The determination of these parameters provides a foundation for the subsequent quantitative analysis of Joule heating effects induced by leakage currents.

To more comprehensively account for the various conduction mechanisms of leakage current in the barium titanate thin film under different electric field strengths, we directly based on the experimental data presented in Wei Zhang et al. [S12] and incorporated the relationship between leakage current density and electric field into the COMSOL simulation model by interpolation. Using this model, we calculated the potential distribution, electric field strength, current density, and Joule heating distribution in the electrically tunable metasurface when a voltage of 10 V is applied to the interdigitated electrodes, with the results shown in **Figure S17**.

The simulation results indicate that upon application of the external voltage, the electric field strength is primarily concentrated in the barium titanate film near the gold interdigitated electrodes. The average electric field strength across the BaTiO₃ film is approximately 130 kV/cm. A distinct interfacial contrast in the electric field distribution is observed at the boundary between the film and the MgO substrate, with further concentration directly beneath the gold electrodes. The leakage current is predominantly confined to a small region directly under the gold electrodes, with a maximum current density of approximately 2.03 × 10⁻⁴ A/cm². **Figure S17(d)** illustrates the Joule heating distribution within the device, revealing that Joule heating is mainly localized within the BaTiO₃ film, particularly in the vicinity of the electrodes. In contrast, Joule heating in the MgO substrate and the a-Si microstructures is negligible.

**Figure S17. Electrostatic response and Joule heating of the electrically tunable metasurface under a voltage of 10 V.** (a) Potential distribution, (b) Electric field intensity distribution, (c) Leakage current density, (d) Joule heat distribution (arrows represent the electric displacement vector).

Received: ((will be filled in by the editorial staff))
Revised: ((will be filled in by the editorial staff))
Published online: ((will be filled in by the editorial staff))

**References**

[S1]	Edward D. Palik, "Handbook of Optical Constants of Solids", ***Academic Press*** (1997).

[S2]	RefractiveIndex.INFO - refractive index database. <https://refractiveindex.info/>.

[S3]	P. B. Johnson and R. W. Christy, "Optical constants of the noble metals", ***Physical Review. B*** 6, 4370-4379 (1972). <https://doi.org/10.1103/PhysRevB.6.4370>.

[S4] S. Fan, W. Suh, and J. D. Joannopoulos, "Temporal Coupled-Mode Theory for the Fano Resonance in Optical Resonators," ***JOSA A***, no. 3 (2003): 569–572. <https://doi.org/10.1364/JOSAA.20.000569>

[S5]	G. Alagappan, "Fabry-Perot Resonances in Bilayer Metasurfaces," ***Physical Review Letters***, no. 22 (2024). <https://doi.org/10.1103/PhysRevLett.133.226901>

[S6]	A. Karvounis, F. Timpu, V. V. Vogler-Neuling, et al., "Barium Titanate Nanostructures and Thin Films for Photonics," ***Advanced Optical Materials***, no. 24 (2020): 2001249. <https://doi.org/10.1002/adom.202001249>

[S7]	P. Castera, D. Tulli, A. M. Gutierrez, and P. Sanchis, "Influence of BaTiO3 Ferroelectric Orientation for Electro-Optic Modulation on Silicon," ***Optics Express***, no. 12 (2015): 15332–15342. <https://doi.org/10.1364/OE.23.015332>

[S8] S. Abel, F. Eltes, J. E. Ortmann, et al., "Large Pockels Effect in Micro- and Nanostructured Barium Titanate Integrated on Silicon," Nature Materials, no. 1 (2019): 42–47. <https://doi.org/10.1038/s41563-018-0208-0>

[S9] C. Wang, R. Wang, X. Cheng, et al., "Passively Broadband Tunable Dual Circular Dichroism via Bound States in the Continuum in Topological Chiral Metasurface," ACS Nano , no. 29 (2024): 18922–18932. <https://doi.org/10.1021/acsnano.4c01697>

[S10] M. Zgonik, P. Bernasconi, M. Duelli, et al. "Dielectric, Elastic, Piezoelectric, Electro-Optic, and Elasto-Optic Tensors of BaTiO3 Crystals," Physical review. B, Condensed matter 50 (1994): 5941–5949. <https://doi.org/10.1103/PhysRevB.50.5941>

[S11] Y. Guo, K. Suzuki, K. Nishizawa, T. Miki, and K. Kato "Thickness Dependence of Electrical Properties of Highly (100)-Oriented BaTiO3 Thin Films Prepared by One-Step Chemical Solution Deposition," Japanese Journal of Applied Physics 45, no. 2R (2006): 855. <https://doi.org/10.1143/JJAP.45.855>
